# Diagnostic Performance of Contrast-Enhanced Ultrasound in Renal Cancer: A Meta-Analysis

**DOI:** 10.3389/fonc.2020.586949

**Published:** 2020-11-18

**Authors:** Ke-Hao Pan, Li Jian, Wei-Jun Chen, Abdul Aziz Nikzad, Fang Q. Kong, Xu Bin, Ya-Li Wang, Ming Chen

**Affiliations:** ^1^ Affiliated Zhongda Hospital of Southeast University, Southeast University, Nanjing, China; ^2^ Department of Urology, Jinhu People’s Hospital, Jinghua, China; ^3^ Department of Urology, JinTan People’s Hospital, Changzhou, China; ^4^ Department of Urology, Affiliated Zhongda Hospital of Southeast University, Nanjing, China; ^5^ Department of Nosocomial Infection, Affiliated Zhongda Hospital of Southeast University, Nanjing, China

**Keywords:** renal cancer, contrast-enhanced ultrasound, diagnosis, meta-analysis, tumor imaging

## Abstract

**Background:**

Contrast-enhanced ultrasound (CEUS) is an examination mode for detecting blood vessels in tissues, and it has been gradually used in the diagnosis of kidney cancer in recent years. This study explores the value of contrast-enhanced ultrasound in the clinical diagnosis of renal cancer, and provides an accurate and effective method for clinical diagnosis of renal cancer.

**Methods:**

CEUS and RCC were selected as the keywords. Searching the PubMed and Embase from 2007 to 2020, the original data were abstracted and performed heterogeneity test with the Meta-Disc software. The weighted sensitivity, specificity, positive likelihood ratio and negative likelihood ratio were calculated, as well as the summary receiver operating characteristic (SROC) curve. Further estimated the diagnostic value of CEUS in the research of renal cancer by calculating the area under the curve (AUC). The quality of evidence in researches was evaluated by QUADAS items. Meta-disc, Review Manager 5.3, and STATA 13 were used.

**Results:**

A total of 20 studies were adopted for Meta-analysis. The weighted sensitivity, specificity, positive likelihood ratio, negative likelihood ratio, and diagnostic odds ratio were 0.97, 0.86, 6.8, 0.04 and 171, respectively; and AUC was 0.97. The results showed that there was high heterogeneity.

**Conclusion:**

CEUS technology has a good diagnostic value for RCC.

## Introduction

Renal cancer (RCC) is the most common primary malignant tumor of the kidney, accounting for 80% to 90% of primary malignant tumors of the kidney (1). In recent years, the incidence of kidney cancer and the number of deaths has increased significantly (2). Most patients with kidney cancer lack typical clinical symptoms and signs at an early stage (3). One third of RCC cases were reported with metastasis by the time of diagnosis (4). Therefore, there is still a need for an effective kidney cancer imaging diagnosis method.

RCC usually presents as a large mass on CT, mostly with soft tissue density; papillary RCC is less malignant than RCC and has less blood supply than its blood supply. Therefore, enhanced CT scan show either uneven or relatively uniform mild to moderate enhancement. CT examination is considered to be a gold standard for the diagnosis of kidney tumors, but CT can easily confuse cystic kidney cancer with renal abscess and hydronephrosis. MRI is usually used as a diagnostic tool for kidney tumors that cannot be characterized by CT, and is mainly used for typical lesions in CT. MRI is also often used in patients who cannot undergo CT enhancement due to impaired renal function. The limitation of MRI is that the acquisition time is long and people with metal implants such as pacemakers cannot be examined. Its availability and timeliness are not as good as CT.

The current clinical diagnosis methods for RCC are mainly imaging examinations such as ultrasound, contrast-enhanced CT, contrast-enhanced MR, non-contrast CT, non-contrast MR among which ultrasound has become the main method due to its simplicity and non-invasiveness, but the accuracy of conventional ultrasound for qualitative diagnosis of tumors is limited. Non-contrast CT/MR can only observe a specific section at a specific time, and the display rate of necrotic lesions is not as good as that of contrast enhanced ultrasound (CEUS), and it may be misdiagnosed due to missing the peak period of tumor enhancement and making the contrast enhancement characteristics unclear.

Contrast-enhanced CT and MR contrast agents can cause certain damage to the physiological functions of the liver and kidneys, and can also cause allergic reactions.

Contrast-enhanced ultrasound is a new detection method developed in ultrasound contrast agent and contrast imaging technology. It can observe blood perfusion in tumor in real time, continuously and dynamically, which further improves the accuracy of clinical diagnosis (5–7). CEUS can effectively display the low blood perfusion state and ischemic necrosis of tumor lesions with a diameter of less than 1 cm, thereby providing more information for the diagnosis of renal cancer, and at the same time eliminates the disadvantages of enhanced CT and MRI examinations (8, 9). CT/MR can only observe a specific section at a specific time. In addition, CEUS can display small blood vessels more sensitively than CT/MR, so as to more accurately observe blood perfusion of new tumors, which can evaluate the angiogenesis of renal cancer before surgery. CEUS cannot observe the surrounding and distant metastasis of the tumor, and cannot provide information on the clinical staging of renal cancer.

This study explores the value of contrast-enhanced ultrasound in the clinical diagnosis of renal cancer. The contrast-enhanced ultrasound examination method has high efficiency in diagnosing kidney cancer, nonradioactive and has very few contrast agents to cause allergic reactions. Compared with MRI, the examination time is short, therefore, it has higher clinical promotion value. With improvement of functions and performance of Doppler ultrasound equipment, the development of safer, cheaper, and better imaging performance contrast agents, contrast-enhanced ultrasound may become the first choice for renal cancer in the near future.

## Methods

### Search Strategy

Computer searches include PubMed, Embase to collect relevant literature on the diagnosis of kidney cancer by contrast-enhanced ultrasound. Search period: 2007 to 2020. Subject terms include contrast-enhanced ultrasound, kidney tumor, kidney cancer, renal cancer and renal tumor, and the search method is adjusted according to the specific database, and the search strategy is determined after multiple pre-searches. Using a combination of database retrieval and manual retrieval, two evaluators independently retrieve and re-search the references of the included literature. Another reviewer Xu Bin and Ke-Hao Pan are both medically-trained urologists in China with certain clinical and imaging experience. Xu Bin is the deputy chief physician of Chinese Urology. The deviation between the two reviewers is relatively small. The language is limited to Chinese or English.

### Study Selection

The exclusion criteria for the systematic review were: (a) articles not within the field of interest; (b) editorials or letters, review articles, comments, conference proceedings; and (c) case reports.

### Literature Screening

Literature was independently screened by 2 reviewers based on the inclusion criteria, first reading the title and abstract. Then read the full text of the documents that may meet the inclusion criteria. After cross-checking the results, data were extracted from cohort studies. The basic characteristics of the included literature are shown in **Table 1**.

**Table 1:** Study and patient characteristics.

Studies	Year	Size Age	Lesions	Study Type	Lesion size	TP	FP	FN	TN
Li et al. (10)	2008	71 53.6	72	Prospective	1.3–5	26	18	0	28
Xu et al. (11)	2010	119 42.9	126	Retrospective	1.5–11.7	82	11	1	32
Lgnee et al. (12)	2010	135 66	127	Prospective	NA	114	11	3	9
Zhou et al. (13)	2011	51 37	51	Prospective	1.5–6	20	14	2	15
Lu et al. (14)	2012	122 41.3	123	Retrospective	1–11.5	105	0	3	15
Li et al. (15)	2013	91 62.0	100	Retrospective	0.9–9.7	83	1	2	14
Oh et al. (16)	2014	49 61	49	Retrospective	<4	33	4	5	7
Barr et al. (17)	2014	721 70	306	Retrospective	0.2–16.1	139	8	0	159
Nicolau et al. (18)	2015	72 64.9	83	Prospective	5–6.5	31	2	2	48
Lu et al. (19)	2015	174 40.3	174	Retrospective	1.0–7.5	136	10	6	22
Chen et al. (20)	2015	99 56.6	102	Prospective	1–3	73	4	17	17
Rubnthaler et al. (21)	2016	36 NA	36	Retrospective	NA	27	0	1	8
Yong et al. (22)	2016	63 48.7	76	Retrospective	0.4–7.9	21	3	1	49
Wei et al. (23)	2017	128 53.6	118	Retrospective	1–3.9	87	8	6	17
Zarzour et al. (24)	2017	41 NA	41	Retrospective	NA	20	3	0	18
Clevert et al. (25)	2008	32 56	37	Retrospective	NA	12	5	0	20
Ascenti et al. (26)	2007	40 48	44	Retrospective	NA	5	0	6	33
Quaia et al. (27)	2008	40 62	40	Retrospective	2–8	18	4	3	15
Guillaume et al. (28)	2017	47 64.7	19	Prospective	1.8–5.8	14	1	0	32
Sanz et al. (29)	2016	66 67.8	67	Prospective	3.8	66	12	0	54

### Quality Assessment

Two reviewers individually evaluated the quality of the included literature, and discussed when they disagree. This meta-analysis was carried out according to the QUADAS (Quality Assessment of Diagnostic Accuracy Studies) standard, which can be divided into three situations: “yes,” “no,” and “unclear.” “Yes” means that the criteria for this item are met, “no” means that the criteria are not met, and “unclear” means that the standards are partially met or sufficient information cannot be obtained from the document (30).

### Statistical Analysis

**Figure 1:**
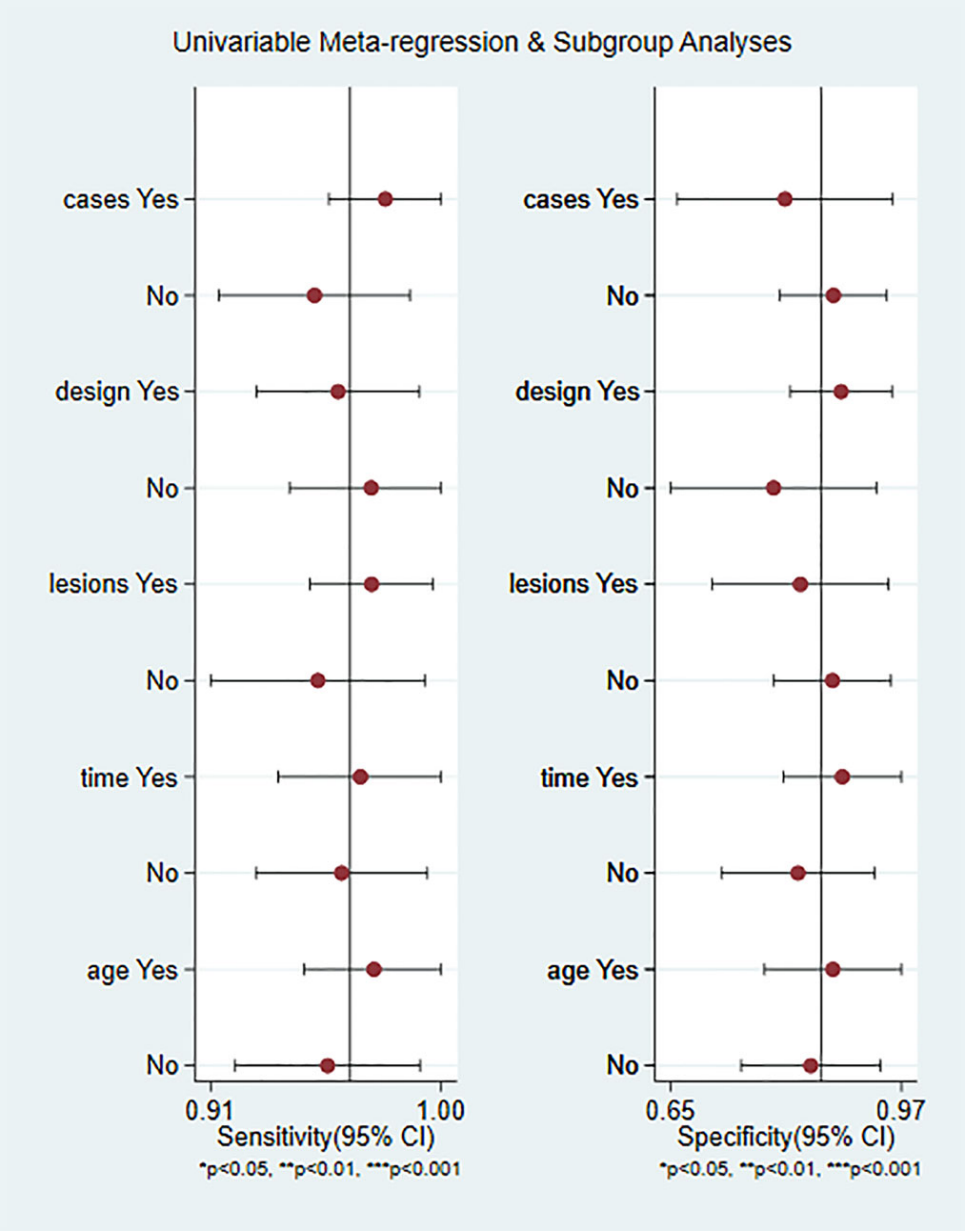
Meta-regression and subgroup analyses of studies.

The weighted sensitivity, specificity, positive likelihood ratio and negative likelihood ratio were calculated, as well as the summary receiver operating characteristic (SROC) curve. The larger the area under the curve and the closer the SROC curve is to the upper left corner, the higher the value of the diagnostic test. Between-study statistical heterogeneity was assessed using I^2^ and the Cochrane Q test. The meta-regression and subgroup analysis of CEUS are shown in **Figure 1**, divided into five sub-groups according to whether the article publication year is beyond 2015, whether the case was greater than 100, whether lesion was greater than 100, whether the study was retrospective or prospective, and whether the age of patients above 60. The number of articles published before and after 2015 is close.

## Results

### Literature Search

**Figure 2:**
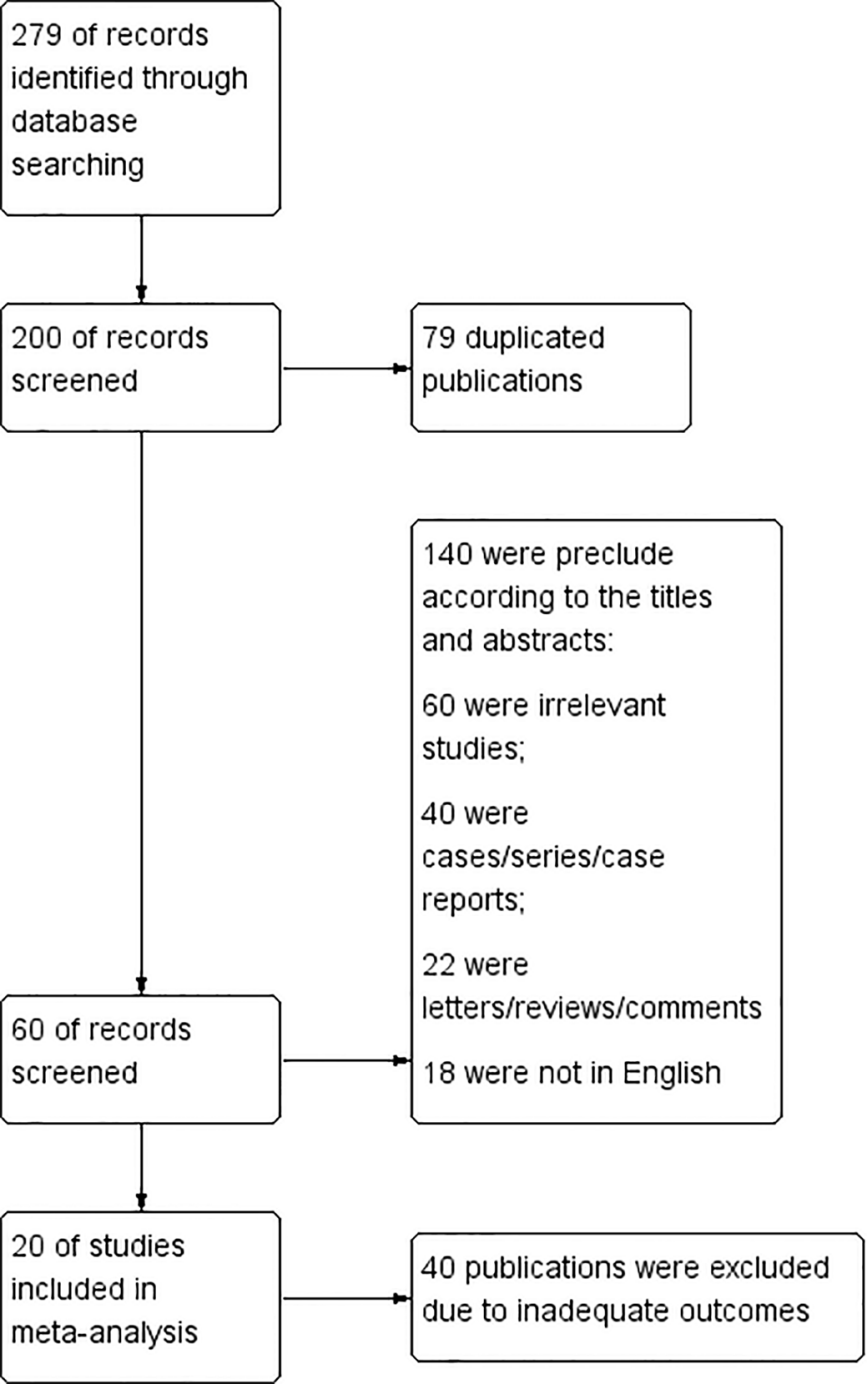
Flowchart.

279 documents were first detected. 79 duplicated publications were excluded through literature manager software. And after the abstracts were screened, 140 records were excluded. 40 publications were excluded due to inadequate outcome because they lack information about the true positive rate, true negative rate, false negative rate and false positive rate of CEUS diagnosis. Finally, a total of 20 articles were included (10–29). The flow chart is shown in **Figure 2**.

Twenty studies including 2197 patients and 1791 lesions were selected for the meta-analysis. The basic characteristics of the included literature are shown in **Table 1**.

Inclusion criteria: 1 Pathological diagnosis should be adopted as “gold standard” for all adopted literature; 2 The research object is the literature using contrast-enhanced ultrasound to diagnose RCC; 3 The interval between ultrasound examination and pathological examination should not exceed 1 month; 4 Each study can be successfully extracted to TP, FP, TN, and FN.

Exclusion criteria: 1 Excluded documents that did not use contrast enhancement technology. 2 Excluded secondary literature and conference papers such as experience exchange, abstracts, lectures and reviews.

### Histopathological Results

The histopathological results of included studies are shown in **Table 2**. Most of the included articles are RCC, Papillary RCC and so on. CEUS is effective in diagnosing these kidney cancers.

**Table 2:** Histopathological results of the included studies.

Author	Histopathological results
Li et al. (10)	RCC
Xu et al. (11)	RCC+Papillary RCC
Lgnee et al. (12)	ccRCC
Zhou et al. (13)	Small Papillary RCC
Lu et al. (14)	RCC
Li et al. (15)	Small Cystic RCC
Oh et al. (16)	Small RCC
Barr et al. (17)	Cystic RCC
Nicolau et al. (18)	RCC+Papillary RCC
Lu et al. (19)	RCC
Chen et al. (20)	RCC
Rubnthaler et al. (21)	Cystic RCC
Yong et al. (22)	RCC
Wei et al. (23)	Papillary RCC
Zarzour et al. (24)	Cystic RCC
Clevert et al. (25)	Small RCC
Ascenti et al. (26)	Papillary RCC
Quaia et al. (27)	RCC+Papillary RCC
Guillaume et al. (28)	Cystic RCC
Sanz et al. (29)	RCC

### Qualitative Analysis

**Figure 3:**
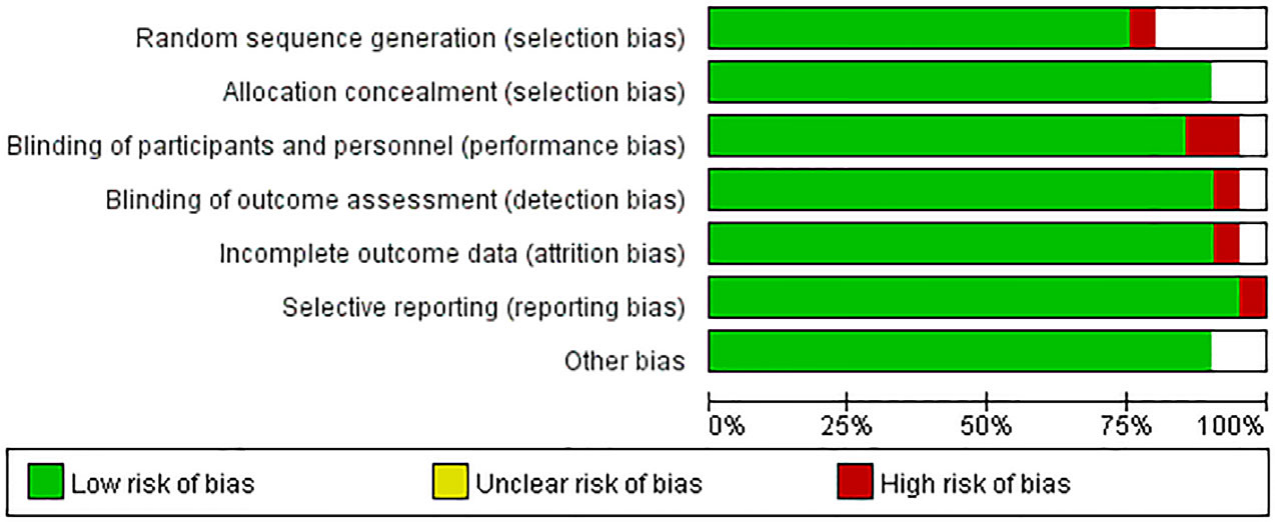
Summary of risk of bias and applicability concerns.

The quality of the articles included was satisfactory. The research quality evaluation is shown in **Figure 3**. In patient selection, one article is high risk. On Index Test, there is no high risk, however, one article on Reference Standard is high risk. The overall quality of the article is high.

### Meta Analysis

**Figure 4:**
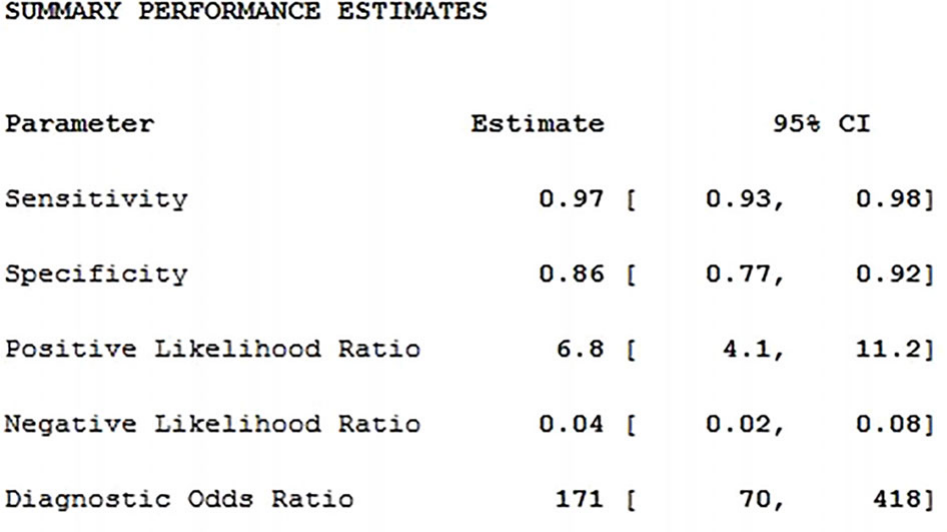
The combined statistics.

**Figure 5:**
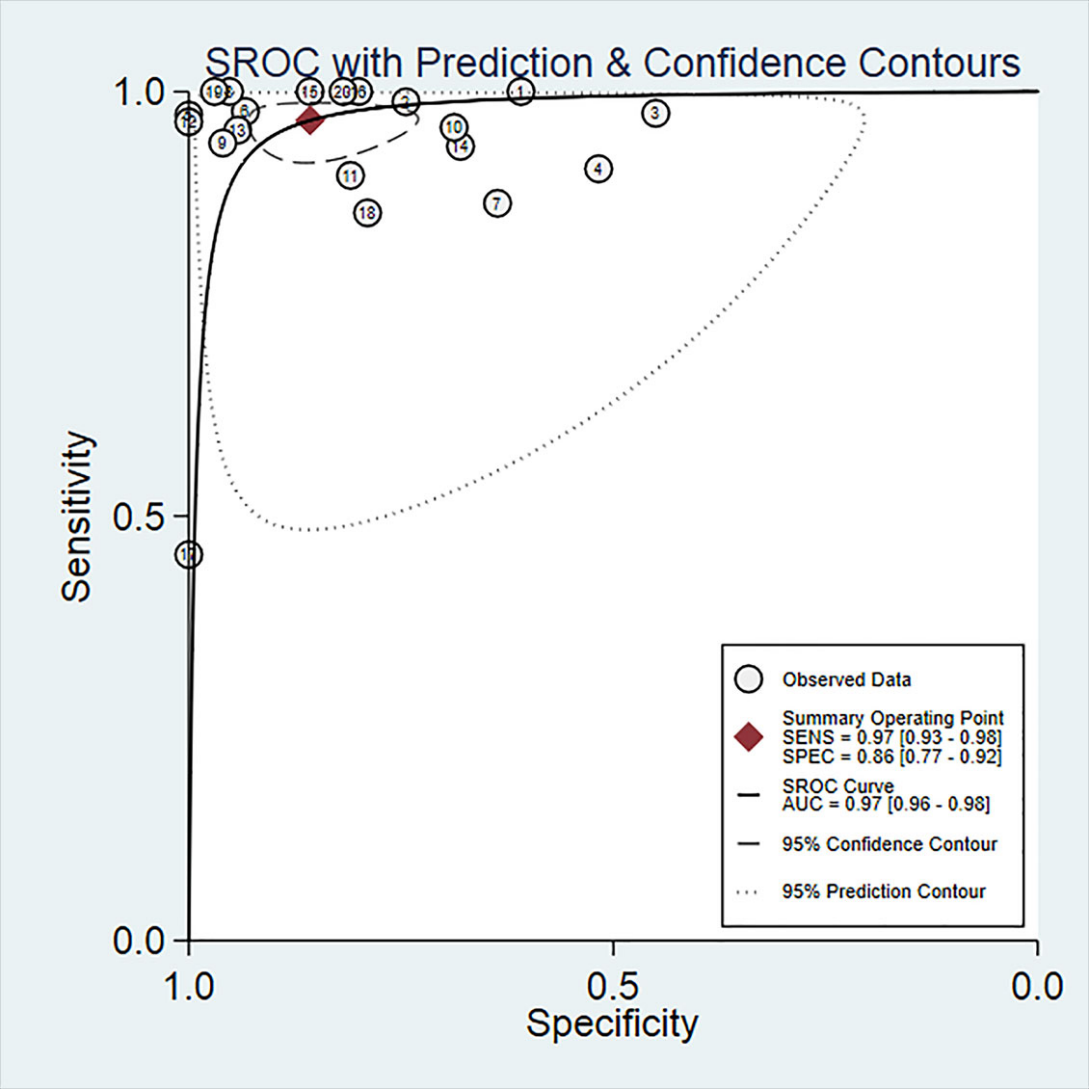
SROC curves of CEUS for diagnosis of renal cancer.

**Figure 6:**
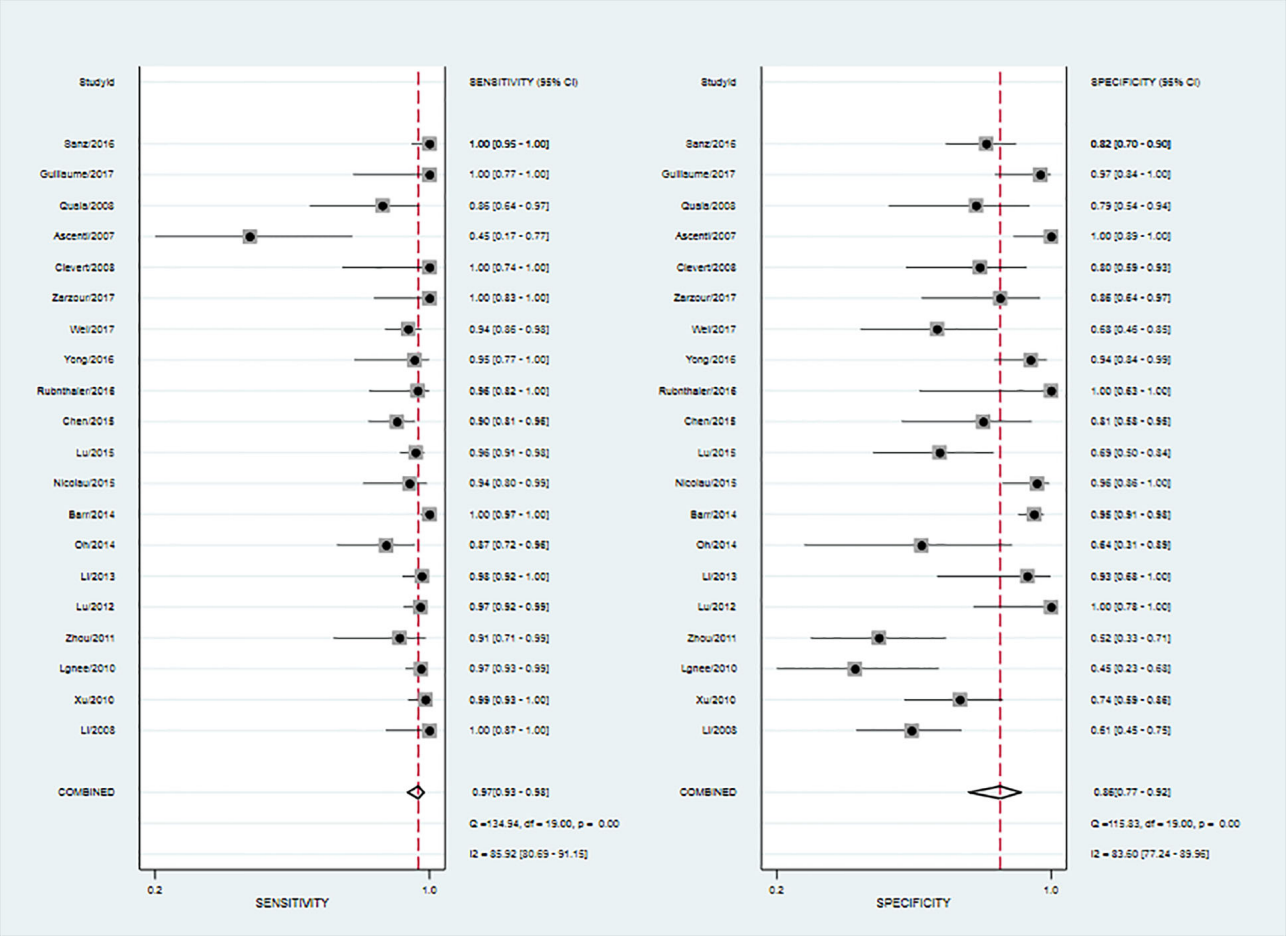
Forest map of CEUS for diagnosis of renal cancer.

Twenty studies including 2197 patients and 1791 lesions were selected for the meta-analysis. Results of the meta-analysis are presented in **Figure 4**. The SROC curve and the forest map of CEUS are shown in **Figures 5** and **6**, respectively. Pooled Sen, Spe, LR+, LR-, DOR were 0.97, 0.86, 6.8, 0.04, and 171, respectively. In **Figure 5**, the areas under the SROC curve are 0.97 (95% CI, 0.96–0.98).

### Heterogeneity Analysis

As shown in **Figure 6**, CEUS has heterogeneity in the sensitivity and specificity of the diagnosis of kidney cancer (Q value, P value, I^2^ value are 134.94, < 0.01, 85.92% and 115.84, <0.01, 83.60%, respectively). A randomed-effects model was used. The Spearman correlation coefficients of the sensitivity logarithm and (1-specificity) logarithm of CEUS diagnosis of renal cancer were −0.190 (P>0.05), indicating that there is no threshold effect. To further explore the potential sources of heterogeneity, a subgroup analysis and meta-regression was performed. It showed that no definite variable was the source of heterogeneity in the current meta-analysis (**Figure 1**).

### Sensitivity Analysis

**Figure 7:**
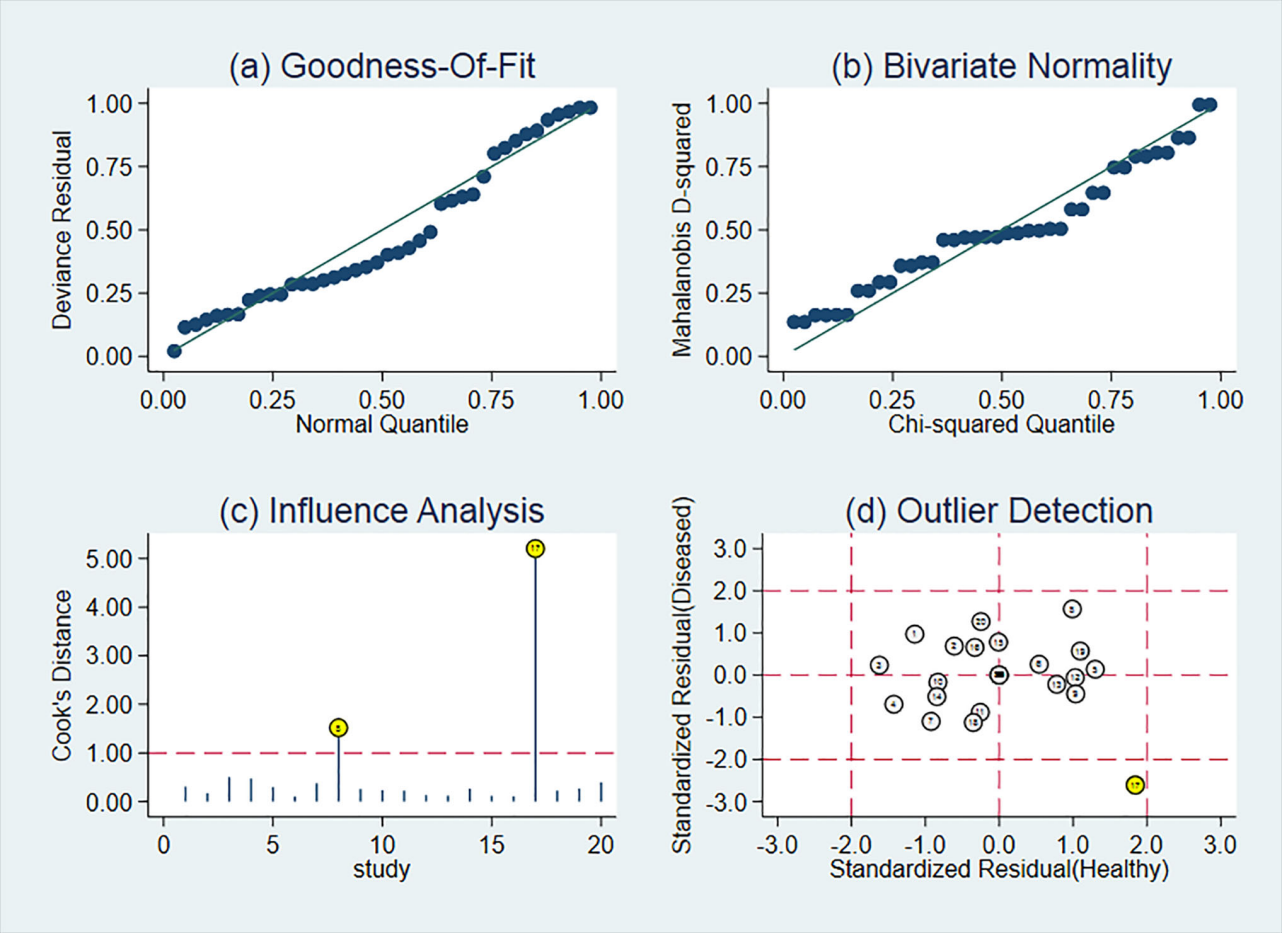
Sensitivity analysis of studies.

Sensitivity analysis is shown in **Figure 7**. The results showed that the meta-analysis results are stable.

### Clinical Application Analysis

**Figure 8:**
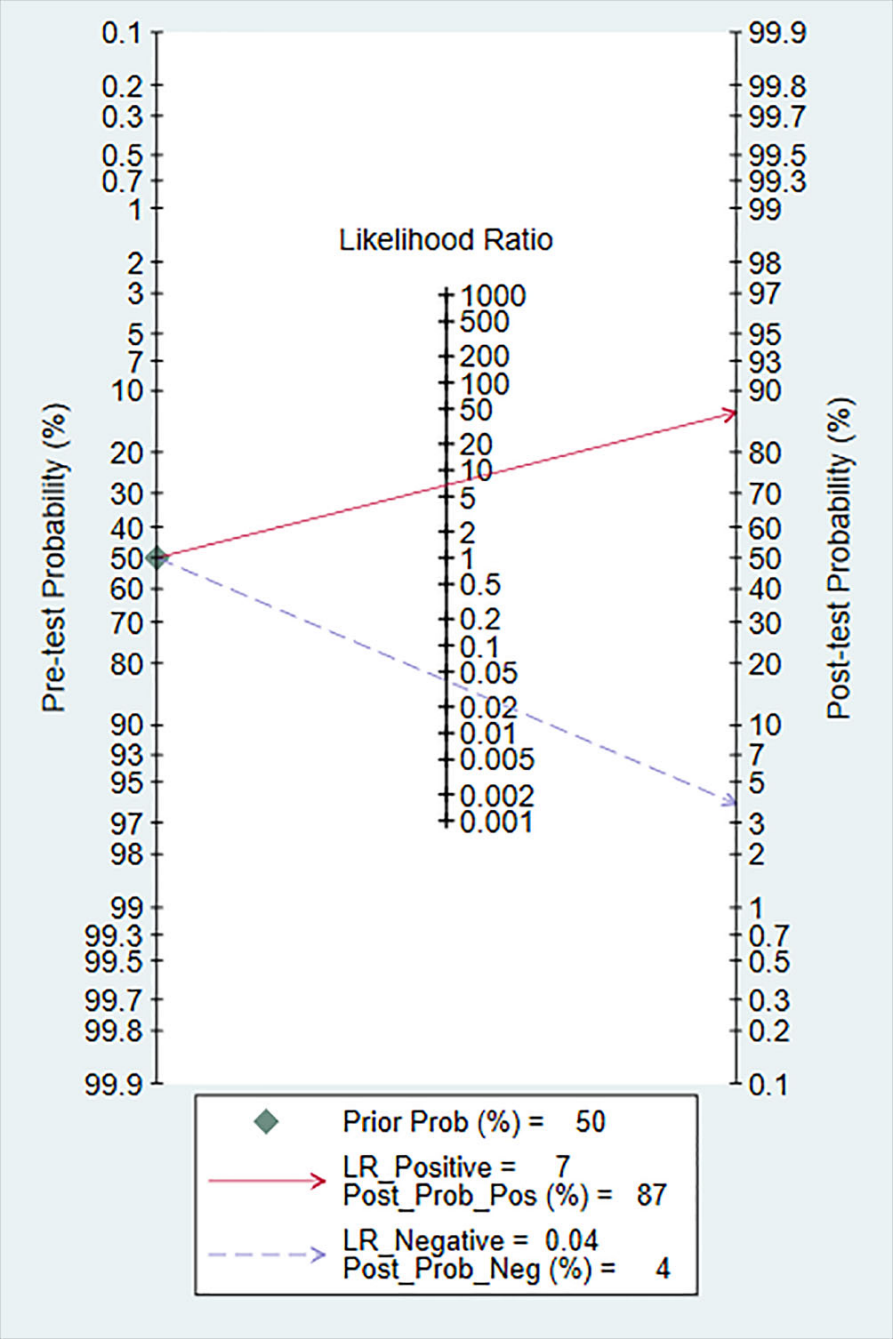
The Fagan map.

**Figure 9:**
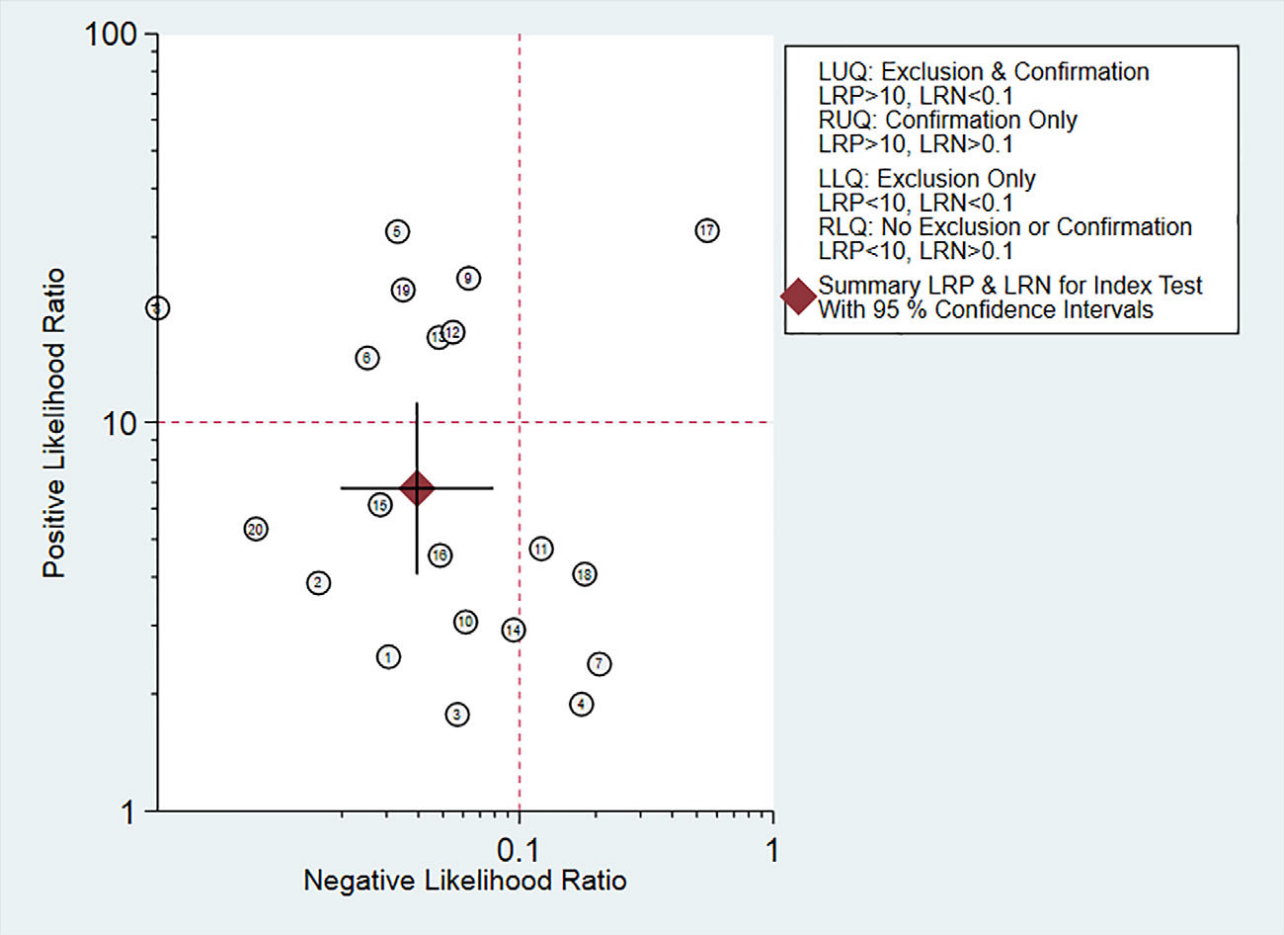
Likelihood ratio dot plot.

Fagan diagram was constructed for clinical application analysis as shown in **Figure 8**. The post-test probability of CEUS was 87% and is higher than the pre-test probability (50%), indicating that CEUS is effective in the diagnosis of renal cancer. As can be seen from **Figure 9**, the combined negative likelihood ratios for the diagnosis of renal cancer were >0.1 and the positive likelihood ratio was <10.

### Publication Bias

**Figure 10:**
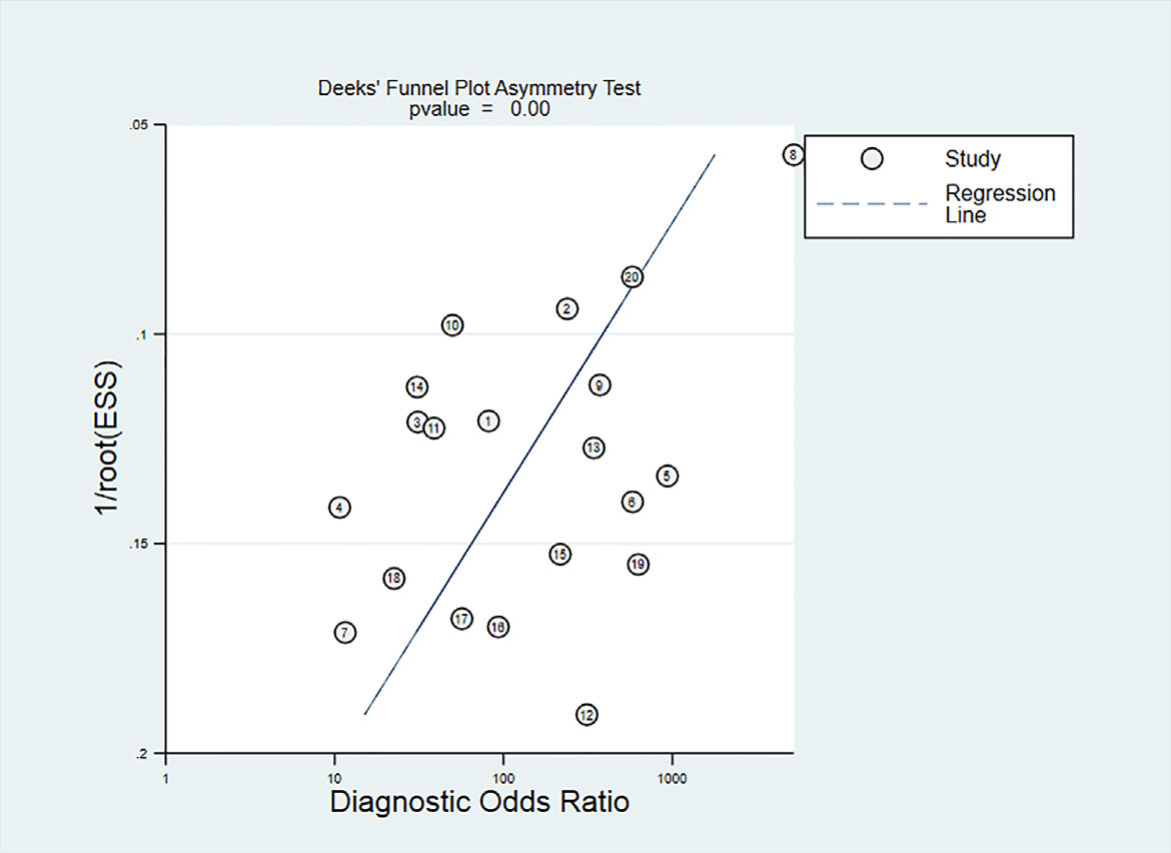
Deeks’ test.

The Deeks’ funnel chart shows asymmetry in scattered points, suggesting that there is publication bias (P<0.05). It is shown in **Figure 10**. However, the sensitivity analysis showed that our results are stable. Despite there is publication bias, our sensitivity test found that the article is stable, indicating that our results are reliable.

## Discussion

CEUS is a new type of ultrasound diagnosis technology that uses contrast enhancers and corresponding analysis software to display the state of tissue blood perfusion on the basis of conventional ultrasound. CEUS uses high-intensity nonlinear harmonic signals generated by contrast agents to increase the contrast between normal tissues and lesions. Kidney cancer has the characteristics of infinite growth of microvessels in malignant tumors. Most malignant tumors have a large number of aggressive capillary formations around and inside the tumor. CEUS can enhance the display of blood perfusion of kidney and tumor microvessels. During the examination, the contrast agent is injected into the blood circulation through the peripheral vein, and the microbubbles are in full contact with the red blood cells in the capillaries, forming many blood bubble interfaces, thereby changing the basic function between the ultrasound and the basic tissues, enhancing the ultrasound signal of the whole body blood pool, and improving the signal-to-noise ratio of echo, thereby improving the display of tumors. In addition, CEUS can also improve the sensitivity of detecting small tumors or slow blood vessels. The results of this study show that the weighted sensitivity, specificity, positive likelihood ratio, negative likelihood ratio, and diagnostic odds ratio were 0.97, 0.86, 6.8, 0.04 and 171, respectively; and AUC is 0.97. This suggests that CEUS could be used as a diagnostic tool for RCC.

CEUS features of renal cancers are that the cortical phase contrast agent can quickly fill the lesion tissue, and the enhancement degree of CEUS is equal to or significantly higher than that of the renal parenchyma, and in the late medulla and delayed phase, the contrast agent quickly exits the lesion organization, mainly manifested as low enhanced performance. CEUS shows the enhancement feature of fast forward and fast out.

CEUS provides a new method for diagnosing kidney tumors. The application of TIC can make the diagnosis of RCC more objective by analyzing the AT, TP, and DPI values of the tumor and surrounding renal cortex. Most renal cancers have pseudo-capsules. The display rate of pseudo-capsules after contrast is higher than that of conventional ultrasound, and the enhancement time is long and obvious. In CEUS, the hemorrhagic and necrotic foci in the tumor are in sharp contrast with the enhanced foci, and the display rate of the necrotic foci is higher than that of conventional ultrasound. Although CT and MRI have high diagnostic rates, contrast agents can cause certain damage to the physiological functions of the liver and kidneys. At the same time, the patient’s body can also be damaged by radiation. CT/MR can only observe a specific section at a specific time, and the display rate of necrotic lesions is not as good as that of CEUS, and it may be misdiagnosed by missing the peak period of tumor enhancement and making the contrast enhancement characteristics unclear. In addition, CEUS can display small blood vessels more sensitively than CT/MR, so more accurate to observe the blood perfusion of new tumors, which can used to evaluate the angiogenesis of renal cancer before surgery. CEUS cannot observe the surrounding and distant metastasis of the tumor, and it has no guidance on the clinical staging of renal cancer.

The heterogeneity of this study is high. According to the results of the subgroup and meta-regression analysis, the five subgroups are not sources of heterogeneity and a comprehensive analysis requires more subgroup data. First, the characteristics of ultrasonography determine that factors such as the experience and skills of the diagnostician, as well as the subjective evaluation of the imaging results, have a great influence on the diagnosis. In Rubnthaler (21) article, all CEUS examinations were performed and interpreted by a single radiologist with more than 15 years of experience in CEUS. In Lu (19) article, a sonologist with 10 years’ experience with CEUS did diagnosis. Obviously, different experiences of the two sonologists will lead to different diagnosis of kidney cancer. Then, although each study uses the same contrast agent, the ultrasound equipment and probe models used are different, and even the same study uses different ultrasound equipment and probes. In Wei (23) article, CEUS examinations were performed using a Sequoia 512ultrasound system (Siemens, Mountain View, CA, USA). In Quaia (27) article, CEUS examinations were performed using Sequoia, Acuson-Siemens. These factors may affect the diagnosis rate and cause heterogeneity. These may be the reason for the heterogeneity.

This research still has some limitations. First, the number of documents is limited and retrospective studies account for a large amount, so can cause selection bias; second, due to the different publication time of the literature, the CEUS diagnostic standards in some literatures have certain differences, which may affect the results; third, There are poor quality research in these documents, which leads to the bias of the publication of this article.

## Conclusion

Contrast enhanced ultrasound technology has a good diagnostic clinical value for RCC.

## Data Availability Statement

The original contributions presented in the study are included in the article/supplementary material. Further inquiries can be directed to the corresponding authors.

## Author Contributions

AN and W-JC revised the manuscript. K-HP, LJ and WJ-C contributed equally to this work and should be considered co-first authors. AN and FK contributed equally to this work and should be considered second authors. XB, Y-LW and MC contributed equally to this work and should be considered corresponding authors. All authors contributed to the article and approved the submitted version.

## Funding

Foundations: This study was funded by The National Natural Science Foundation of China (No. 81872089, 81370849, 81672551, 81300472, 81070592, 81202268, 81202034), Natural Science Foundation of Jiangsu Province (BK20161434, BL2013032, BK20150642 and BK2012336), Six talent peaks project in Jiangsu Province, Jiangsu Provincial Medical Innovation Team (CXTDA2017025), The National Key Research and Development Program of China (SQ2017YFSF090096), Jiangsu Provincial Key Research and Development Program (BE2019751), Innovative Team of Jiangsu Provincial (2017ZXKJQWO7), Jiangsu Provincial Medical Talent (ZDRCA2016080).

## Conflict of Interest

The authors declare that the research was conducted in the absence of any commercial or financial relationships that could be construed as a potential conflict of interest.

## Abbreviations

RCC, renal cell carcioma; CEUS, contrast-enhanced ultrasound; AUC, area under the curve.
